# How institutional quality, and energy production sources, affect the environmental sustainability of bri countries: A comparison of different income groups

**DOI:** 10.1371/journal.pone.0291144

**Published:** 2023-09-12

**Authors:** Weiyan Sheng, Fei Meng, Muhammad Waqas Akbar

**Affiliations:** China Center for Special Economic Zone Research, Shenzhen University, Shenzhen, Guangdong, China; National University of Sciences and Technology, PAKISTAN

## Abstract

Institutions and energy production sources shape environmental policies and practices. Institutions establish frameworks for renewable energy and enforce environmental protection measures. Conventional energy sources cause pollution and climate change, while green energy sources have lower environmental impacts. In this study we analyzed how quality institutions, along with different types of energy production sources affect the quality of environment in 101 countries that are part of the BRI, a global development project. We used a statistical method called panel quantile regression to analyze data from 2000 to 2020. We found that producing energy from renewable sources, such as wind and solar, reduces CO_2_ emissions by 0.003% in BRI countries. However, producing energy from non-renewable sources, such as gas and coal, harms the environment more in high-income and middle-income countries. We also found that having better institutional quality reduces CO_2_ emissions by 3.421%, 2.710%, and 0.006% in different groups of BRI countries. This means that having stronger and fairer institutions can help protect the environment by limiting the use of non-renewable energy sources and encouraging the use of renewable ones. Our study suggests that improving institutional quality is a key factor for achieving green energy and environmental sustainability in BRI countries.

## Introduction

The natural environment is degrading at an alarming rate, which is a major economic concern for both economists and environmentalists. The rapid increase in carbon dioxide (CO_2_) emissions has caused many serious problems for the world, such as climate change, natural disasters, and low living standards. [1] argue that both industrialized and developing countries have faced more environmental challenges in recent years, as CO_2_ emissions have adversely affected the natural conditions of the environment. According to a report by British Petroleum, per capita CO_2_ emissions in Organization for Economic Co-operation and Development (OECD) countries have decreased by 1%, while emissions from non-OECD countries have increased by 62.4% [2]. Various factors contribute to environmental degradation, but one of the main sources of CO_2_ emissions is the use and production of traditional energy, along with unsustainable human activities. Most countries in the world depend on fossil fuels for their industrial output and economic activity. The IPCC (Intergovernmental Panel on Climate Change) reports that more than 80% of energy has been produced by using fossil fuels in underdeveloped and developing countries [3]. [4] state that droughts, high temperatures, floods, water contamination, sulfur and methane emissions, and CO_2_ emissions are environmental threats to developing countries. In view of these threats, all countries are trying to shift to green energy or at least an energy-mix [5, 6].

**Fig 1:**
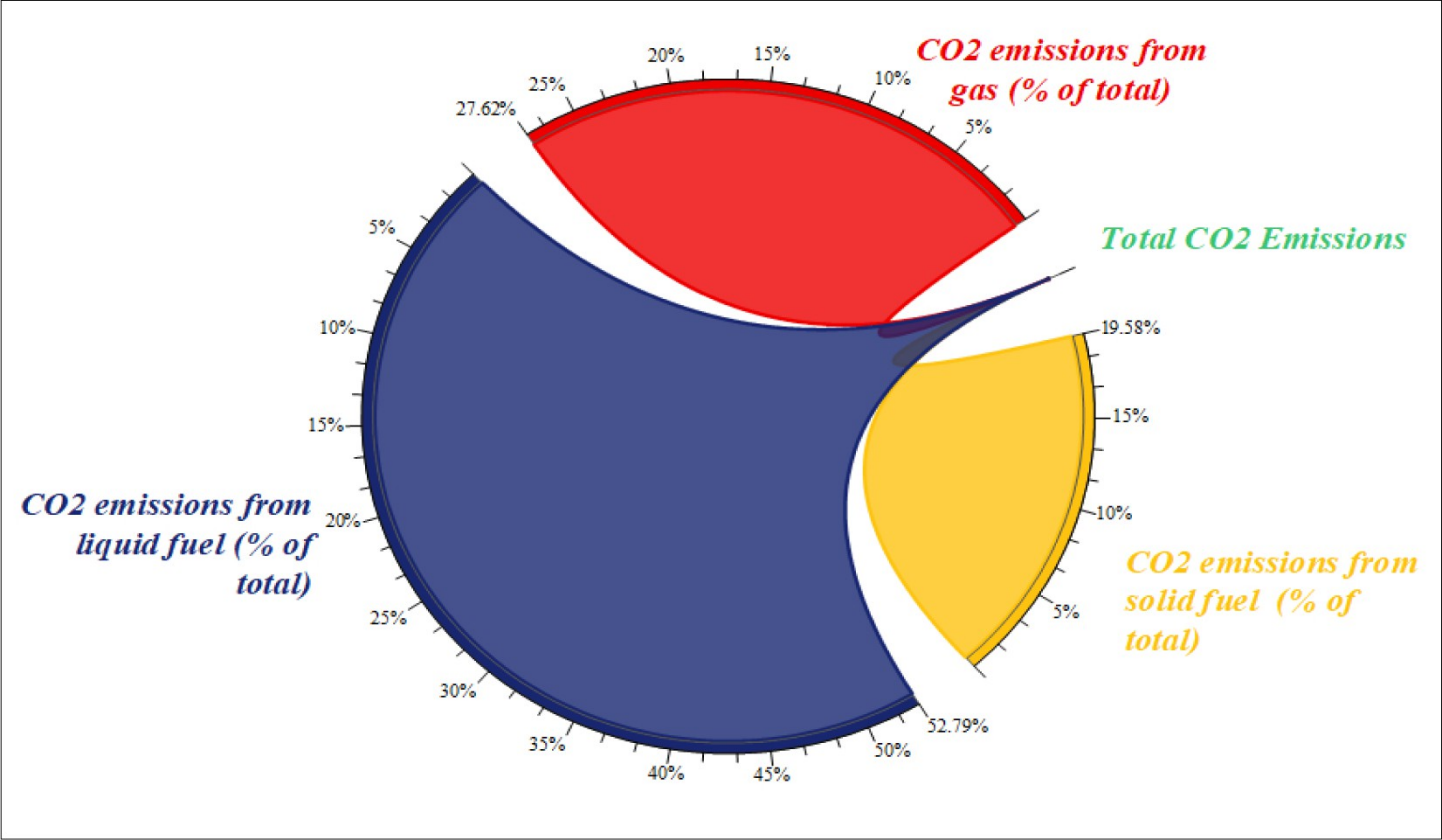
Percentage of CO_2_ emissions from different sources in BRI countries. Source: Author’s own collection by using Origin software (WDI, 2021).

**Fig 2:**
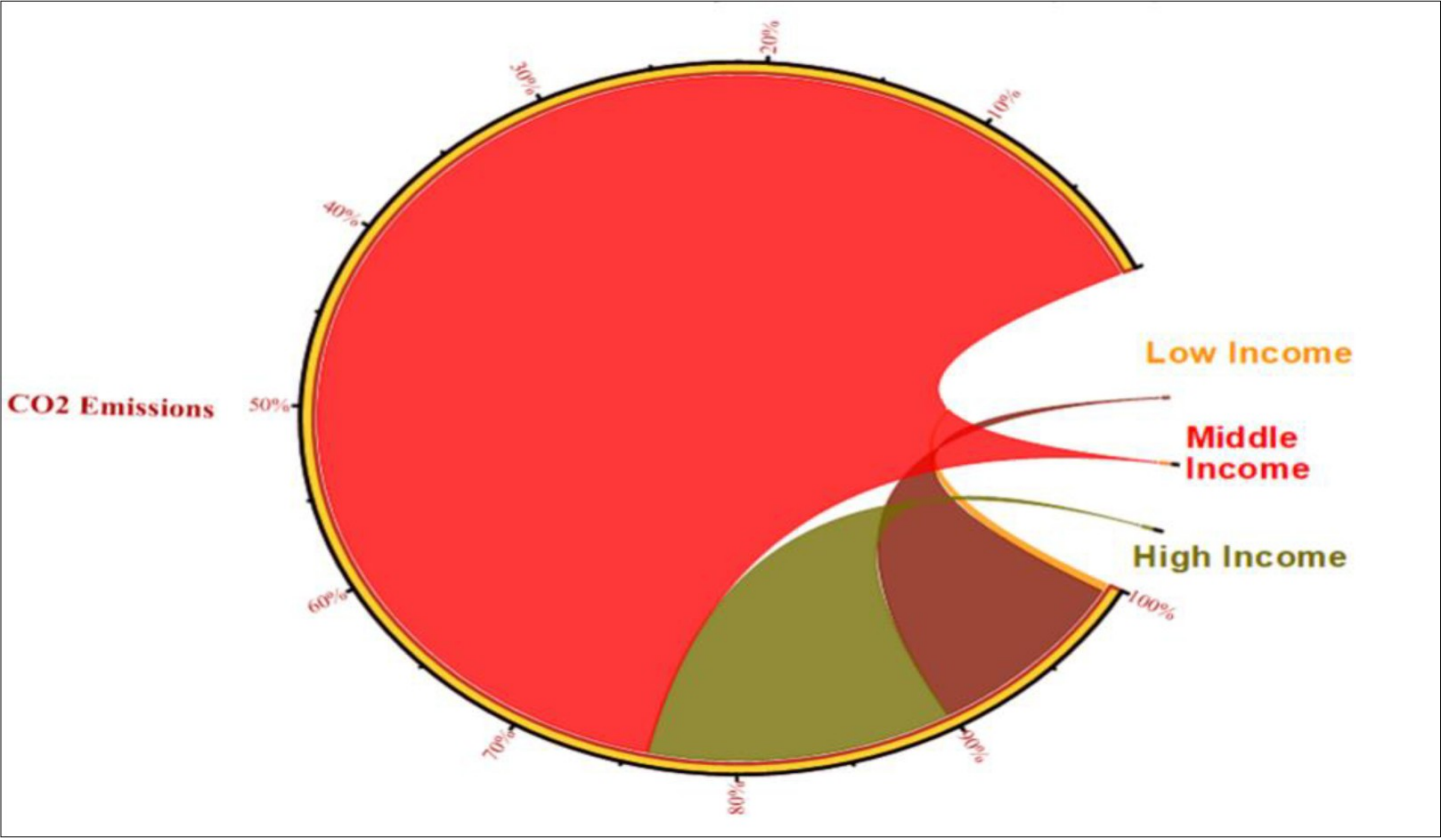
Percentage of CO_2_ in income-wise BRI countries. Source: Author’s own collection by using Origin software (WDI, 2021).

Fig 1 shows the breakdown of CO_2_ emissions by different sources in the Belt and Road Initiative (BRI) region. The three main sources are gas (27.6%), liquid fuels (almost 58%), and solid fuels (19.58%). Fig 2 compares the CO_2_ emissions in the BRI region by income level and reveals that middle-income countries emit the most CO_2_ in the region, followed by high- and low-income BRI economies.

**Fig 3:**
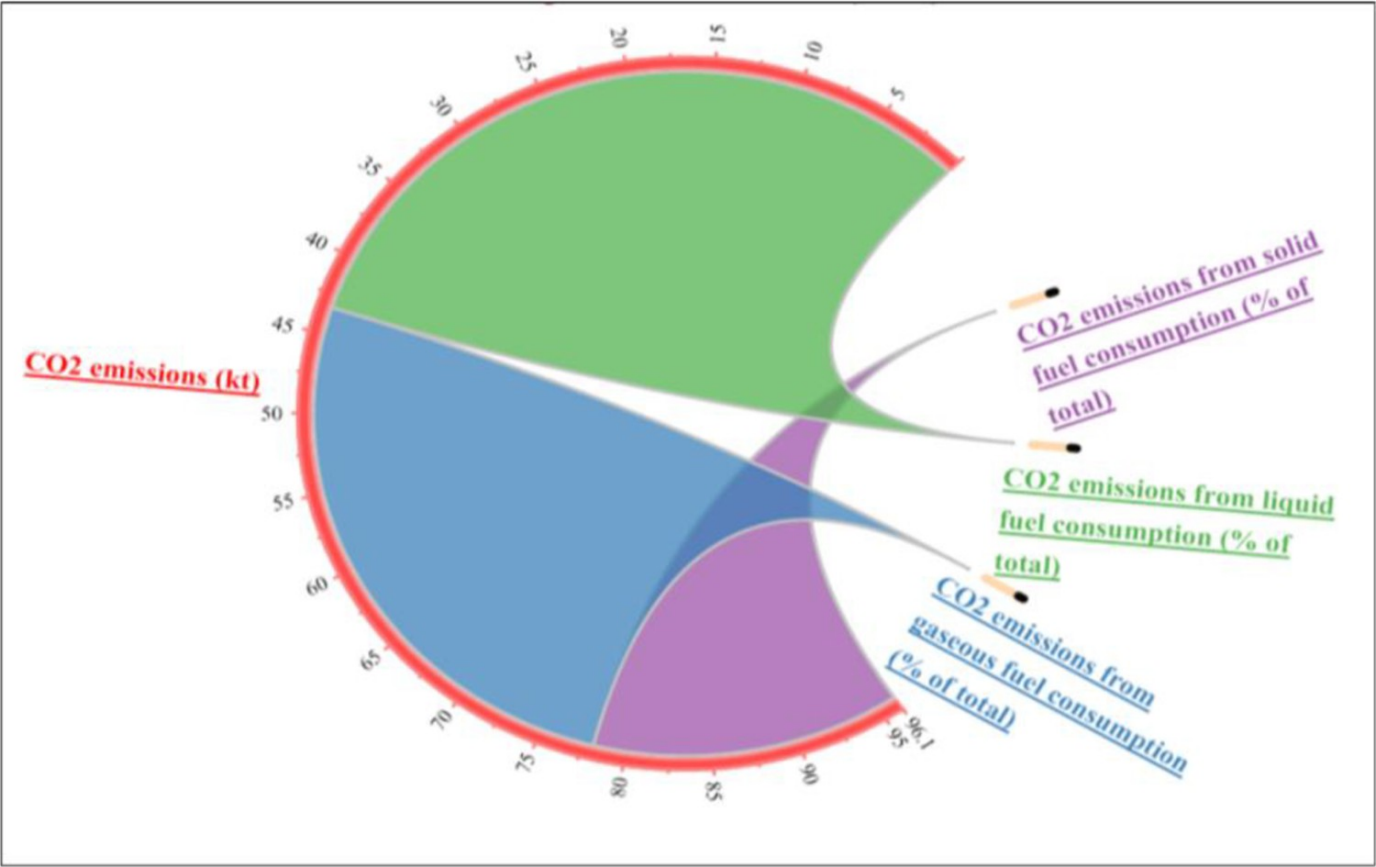
Share of CO_2_ emission sources in high-income countries of the BRI region. Source: Author’s own collection by using Origin software (WDI, 2021).

**Fig 4:**
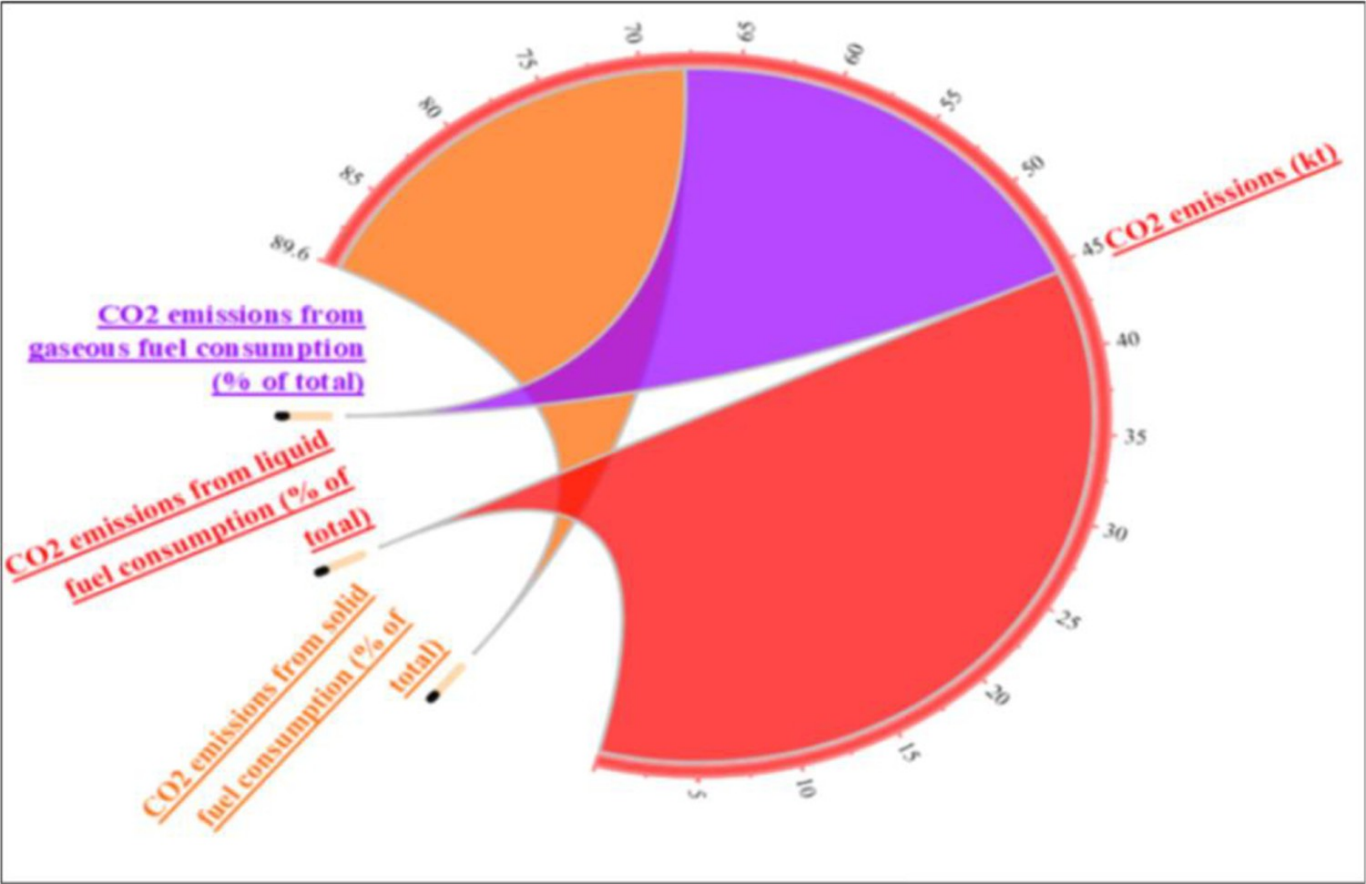
Share of CO_2_ emission sources in middle-income countries of the BRI region. Source: Author’s own collection by using Origin software (WDI, 2021).

**Fig 5:**
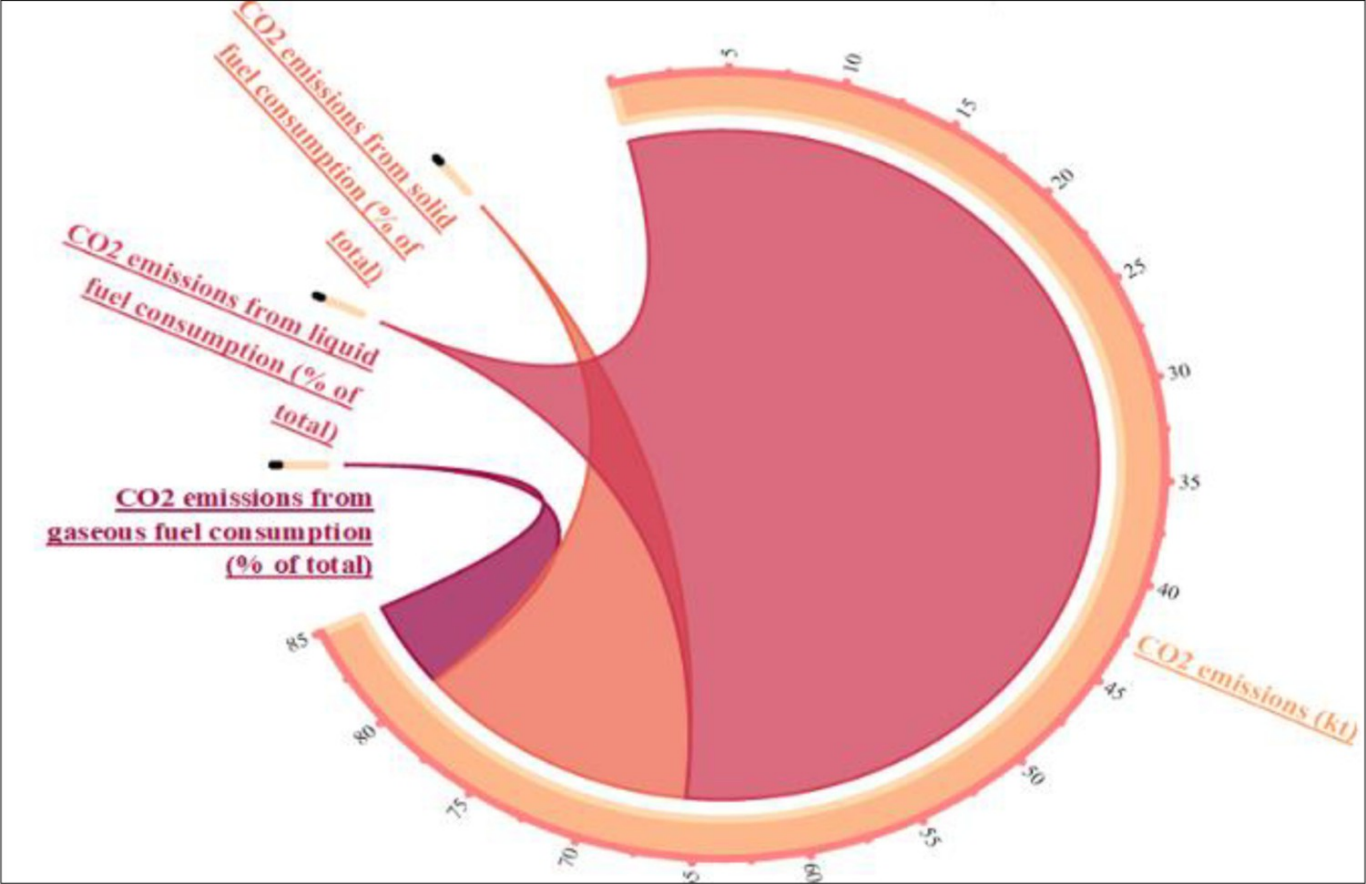
Share of CO_2_ emission sources in low-income countries of the BRI region. Source: Author’s own collection by using Origin software (WDI, 2021).

Fig 3 illustrates the sources of CO_2_ emissions in high-income countries. The majority of CO_2_ emissions come from gas and liquid fuels, while solid fuels contribute to nearly 20% of the total. Figs 4 and 5 depict the sources of CO_2_ emissions in low- and middle-income countries. Liquid fuel consumption is the main source of CO_2_ emissions in both groups, with about 45% in middle-income and 65% in low-income countries. Gas and solid fuels have similar shares of CO_2_ emissions in middle-income countries, but in low-income countries, solid fuels account for only 15% and gas for the rest.

Switching from traditional to green and clean energy is essential for environmental sustainability. However, this transition is not easy for all countries, especially low- and middle-income ones. This poses a dilemma because investing in R&D and renewable energy depends on economic growth. And since cutting down on fossil fuels would hurt their economies, developing countries find it hard to increase the production and share of renewable energy in their total energy mix [6]. Furthermore, institutional challenges in developing countries also affect the energy sector and influence the choice and production of energy from fossil fuels to alternative sources [4, 7, 8].

Institutional quality (IQ) is a broad term that covers the rule of law, individual rights, and excellent public services. Institutional quality and economic growth mutually reinforce each other in the long run. Since the beginning of this century, countries with strong institutional quality have been more successful in adopting advanced technology and enhancing productivity [9]. The government faces institutional challenges that threaten the long-term sustainability of the environment [10]. [11] found that quality institutions helped lower environmental degradation in developed countries. Authorities govern the affairs of countries to achieve short-, medium-, and long-term goals through good initiatives and policies for sustainable development. The government of each country is responsible for creating policies that will enable them to achieve their goals most effectively and efficiently.

The quality and effectiveness of institutions depend on how well they implement and oversee policies. The level of corruption and external influence in institutions affects how well they perform their functions. Institutional quality can be assessed by certain standards, and laws are enforced to prevent and address violations. Institutions are responsible for creating, strengthening, and executing economic policies. The policies can either promote or discourage behaviors that affect the nation’s development [12]. Moreover, [13] found that emerging countries have less strict environmental rules and regulations, suggesting that political factors may act as a barrier to environmental quality [14]. A country’s institutions are more likely to implement policies poorly if they are dominated by a political leader or corrupted by dishonest practices. Bribery, rule-breaking, tax evasion, process skipping, and penalty dodging are some examples of corrupt actions that are facilitated by weak and corrupt institutions. Despite the continuous deterioration of the environment, some countries have achieved significant economic growth due to the role of IQ in economic development [12].

Many studies have examined the relationship between energy consumption policies for both renewable and non-renewable sources, economic growth, and other factors [15–24]. However, none of these studies have considered the role of institutional quality in BRI and its subgroups by income level. This study fills this gap in the literature with six contributions. First, unlike previous studies that analyzed the effects of traditional, renewable energy sources and institutional quality on the environment separately, this study examines these effects together. Second, this study uses a panel of up to 101 BRI countries. The main reasons for choosing the BRI region for this study are as follows: The initiative involves 39 countries from sub-Saharan Africa, 34 from Europe and Central Asia, 25 from East Asia and the Pacific, 18 from Latin America and the Caribbean, 17 from the Middle East and North Africa, and 6 from South Asia. BRI is a global initiative that faces various challenges due to the lack of relevant policies. It is important to address issues related to technologies, climate change, governance, and energy sector regulations.

Climate change is a major concern as it causes severe natural disasters. BRI countries account for about 65% of global CO_2_ emissions (IEA, 2018). China has invested 769 billion US dollars in this initiative, and 39% of this amount will go to energy-related projects, 26% to the transport sector, and 7% to metal [25]. The BRI region has 58.5% of the world’s crude oil reserves and produces 53.8%, 74.69%, and 55.17% of the world’s natural gas, coal, and oil, respectively [1, 26]. The BRI region also comprises 62% of the world’s population, 31% of the world’s GDP, and 35% of the world’s trade.

This study makes several novel contributions to the existing body of research, aiming to strengthen the connection between the dependent and independent variables. Firstly, it assesses the influence of institutional quality and disaggregated energy production sources, including electricity generated from oil, natural gas, coal, and renewables, on CO_2_ emissions. This examination is particularly significant in emerging economies where poor institutional quality may impede the adoption and enforcement of environmental laws, exacerbating energy policies and environmental issues. Secondly, to gain a comprehensive understanding and propose tailored policies, this study divides the panel into three sub-panels: high-income, middle-income, and low-income countries. By considering the income levels of the countries, the effects of the aforementioned variables can be examined and policy recommendations can be formulated accordingly. It is widely acknowledged that production and environmental policies vary based on a country’s income level. Moreover, this study employs advanced panel quantile regression to analyze the impacts of these factors on CO_2_ emissions. By utilizing this statistical method, a deeper understanding of the relationship between the variables and CO_2_ emissions can be obtained, accounting for different quantiles of emissions and providing valuable insights. Finally, this study aims to provide policy recommendations for countries involved in the BRI as well as countries with varying income levels, particularly regarding the transition from traditional to renewable energy production. Additionally, it will suggest policies pertaining to institutions, emphasizing how high-quality institutions can enhance environmental quality and facilitate a transition towards sustainability.

## Literature review

### Institutional quality and environmental

Institutional quality, which encompasses respect for individual rights, the rule of law, and the provision of high-quality public services, plays a crucial role in the long-term development of economies. This strong institutional quality has enabled countries to embrace advanced technologies and enhance productivity since the early 2000s [9]. However, governments often encounter institutional challenges that pose threats to the long-term sustainability of the environment [10]. Interestingly, the improvement of institutional quality in developing countries has also stimulated widespread technological progress in developed nations, leading to increased global output [27]. The concept of institutional quality refers to a set of rules that regulate social, political, and economic interactions, as well as individual behaviors [28].

Studies has found a positive relationship between institutional quality and environmental quality. For instance, [4] investigated the effect of institutional quality on energy production and environmental pollution in 66 emerging nations. Their findings suggested that higher institutional quality improves energy efficiency and reduces CO_2_ emissions in a globalized world. Similarly, [29] explored the role of high-quality institutions in enhancing environmental quality across 47 developing countries, revealing a positive association between institutional quality and environmental outcomes. It is worth noting that various studies have employed different measures of IQ. [11] for example, analyzed the influence of governance on CO_2_ emissions in Brazil, Russia, India, China, and South Africa (BRICS) countries over a 21-year period. They argued that better governance leads to reduced CO_2_ emissions and minimized environmental harm.

Several studies have consistently shown a connection between institutional quality and CO_2_ emissions [26]. For instance, [30] conducted an empirical evaluation of how institutions shape environmental quality in South Asian economies and found a favorable effect. Similarly, [14] demonstrated that IQ plays a role in limiting CO_2_ emissions in Sub-Saharan African countries. The specific measures used to assess institutional quality vary across studies. [4] discovered in their research that IQ positively influences energy production and reduces environmental pollution in 66 emerging nations. They emphasized the importance of quality institutions in controlling CO_2_ emissions in the context of globalization. Likewise, [29] explored 47 developing countries and found that quality institutions contribute significantly to improving environmental quality. Their study highlighted the positive association between IQ and environmental outcomes.

In the context of governance and CO_2_ emissions, [11] examined data from BRICS countries spanning from 1996 to 2017. Their findings suggested that better governance is associated with reduced CO_2_ emissions, leading to a decrease in environmental harm. It is important to note that other studies have also demonstrated a link between institutional quality and CO_2_ emissions [26]. [30], for example, empirically assessed the contribution of institutions to enhancing environmental quality in South Asian economies and discovered a positive impact.

[31] conducted a study exploring the impact of IQ on the environment, specifically in Middle East and North African (MENA) countries. They argued that corruption significantly deteriorates environmental quality. The study focused on the relationship between institutional quality, corruption, the shadow economy, and environmental pollution, utilizing data from over 100 countries spanning the period from 2004 to 2007. The findings revealed that MENA countries exhibit higher pollution levels, and both corruption and the shadow economy contribute to increased emissions. This is primarily due to the detrimental effects of corruption and the shadow economy on the effectiveness of governmental institutions, rendering pollution control measures less enforceable. For instance, firms operating without proper licenses or registrations may engage in corruption or operate within the shadow economy to evade regulations and taxes [32]. In a similar vein, [33] analyzed the relationship between pollution and corruption across 94 countries from 1987 to 2000. The study discovered that corruption has a negative impact on environmental outcomes as it leads to environmental degradation. Whereas, [34] found in his study that Institutional quality plays a significant role in shaping CO_2_ emissions in Sub-Saharan Africa (SSA) countries. The study revealed that political stability, government effectiveness, democracy, and control of corruption have a negative impact on CO_2_ emissions. These institutional factors contribute to reducing emissions by promoting effective governance and environmental regulation. However, it is worth noting that regulatory quality and the rule of law show a positive effect on CO_2_ emissions, suggesting that certain aspects of institutional quality may unintentionally contribute to increased emissions.

Institutional quality plays a crucial role in economic development and the long-term sustainability of the environment. It encompasses aspects such as respect for individual rights, the rule of law, and the provision of high-quality public services. While research on the relationship between institutional quality and environmental quality has yielded mixed results, several studies have identified positive associations between institutional quality and improved environmental outcomes. Moreover, multiple studies have consistently demonstrated a link between institutional quality and CO_2_ emissions, indicating the beneficial role of quality institutions in shaping environmental quality in various regions. Additionally, research has shown that corruption and the shadow economy have detrimental effects on environmental quality, particularly in MENA countries, by undermining governmental institutions and hindering pollution control measures. These findings highlight the significance of addressing corruption and promoting quality institutions to enhance environmental outcomes on a global scale.

### Disaggregated energy sources and environmental

The relationship between the environment, energy production, and consumption has received significant attention in the existing literature due to its importance. Several studies, including [1, 7, 35–37] have consistently found that traditional energy use contributes significantly to environmental degradation. In contrast, adopting green and clean energy sources is crucial for reducing CO_2_ emissions and protecting the environment. However, it is essential to consider the income levels of countries when implementing energy, environment, and institutional policies. [38] conducted research that demonstrated how generating electricity from renewable sources can effectively lower carbon emissions in African countries. Additionally, the study highlighted that electricity production from fossil fuels, such as oil, natural gas, or coal, leads to higher carbon emissions compared to hydropower generation. Therefore, to achieve sustainable development and reduce carbon emissions in African countries, both energy consumption and production need to be considered.

Further supporting these findings, [39] confirmed the positive impact of renewable energy in decreasing carbon emissions. However, they emphasized that simply increasing renewable energy adoption and real income levels may not be sufficient to address pollution effectively. To address this, they recommended that governments in these countries should adopt policies and laws that actively promote the use of renewable energy. [40] conducted research that revealed the adverse impact of economic growth, energy use, and non-renewable energy sources on environmental damage. However, the study also identified that clean energy adoption, foreign direct investment, and free trade can mitigate such damage. Additionally, the study found that the Environmental Kuznets Curve (EKC) hypothesis, which suggests an inverted U-shaped relationship between income and environmental degradation, is less applicable when China is included in the Association of Southeast Asian Nations (ASEAN) panel. In the ASEAN region, financial development was found to reduce the negative effects of energy use on the environment, whereas in both ASEAN and China, it exacerbated these effects.

[41] made predictions indicating that China would surpass the United States in renewable energy production by 2026 while also significantly reducing its CO_2_ emissions. [42] demonstrated that traditional energy consumption contributes to economic growth but also leads to higher CO_2_ emissions. [43] uncovered a negative relationship between renewable energy consumption and CO_2_ emissions across eighty-five industrial countries. [44] examined the impact of energy use and trade openness on CO_2_ emissions and discovered a positive and significant effect of energy use on emissions. They concluded that increasing energy use would further deteriorate environmental quality by elevating CO_2_ emissions. [45] demonstrated a bidirectional causality between energy consumption and CO_2_ emissions.

Furthermore, [1] found a long-term relationship between oil, coal, and gas production and CO_2_ emissions, highlighting their positive and significant effects. They emphasized the necessity of reducing energy production from these sources to effectively address climate change. [46] explored the causal relationship between real output, non-renewable energy consumption, renewable energy consumption, and CO_2_ emissions in the BRICS countries from 1992 to 2013. Additionally, [5] identified that increasing energy efficiency can lead to a reduction in CO_2_ emissions in the long run, emphasizing the importance of energy efficiency measures in mitigating emissions. In a recent study by [47] it was found that neural networks outperform econometric models in predicting crude oil prices. These findings have significant implications for governments, corporations, investors, and energy-dependent economies. The research highlights the challenges of price prediction and emphasizes the impact of various factors. Additionally, renewable energy development heavily relies on essential resources, particularly minerals, which are crucial for production [48]. [49] investigated the influence of mineral resource trading on renewable energy development using data from 30 Chinese provinces. Their study emphasizes the need to increase the proportion of renewable energy in the energy mix and enhance mineral reprocessing rates to support renewable energy capacity investments. Furthermore, [50] analyzed the relationship between hydropower energy usage and CO_2_ emissions in different countries, revealing that hydropower reduces emissions in most economies, except for Turkey. This finding highlights the importance of careful consideration in formulating hydropower and environmental sustainability policies. The role of financial development in addressing energy poverty is emphasized by [51], who recommend focusing on improving financial development to alleviate energy poverty. [52] analyze the role of green finance and digital finance in environmental protection and decarbonization. Their findings demonstrate that green finance, renewable energy investment, and technological innovation contribute to reducing CO_2_ emissions. Moreover, [53] explored the relationship between energy usage reduction, green total factor productivity (GTFP), and artificial intelligence (AI). Their study reveals that AI affects carbon intensity differently across sectors and development stages, with labor- and technology-intensive sectors showing a greater potential to reduce carbon intensity.

The reviewed studies consistently highlight the negative impact of traditional energy use on the environment, emphasizing the urgent need to transition towards green and clean energy sources. However, the income levels of countries must be considered when formulating energy, environment, and institutional policies. Previous studies have not thoroughly examined the relationship between IQ and climate change, nor have they adequately assessed the impacts of different energy production sources. Therefore, this study aims to fill this research gap by investigating the effects of energy production from coal, gas, oil, and renewables, as well as institutional quality, on CO_2_ emissions in the BRI regions and income-wise disaggregated BRI countries. Employing the innovative econometric analysis technique known as Sim and Zhou’s quantile-on-quantile (Q-Q) regression, this study provides robust empirical insights beyond conventional methods such as ordinary least squares (OLS) and quantile regression. The Q-Q approach enables policymakers to identify energy sources that effectively reduce CO_2_ emissions within the BRI context. The findings of this study will offer valuable guidance to policymakers in making informed decisions about energy production sources and reducing CO_2_ emissions in the BRI regions.

### Theoretical background

The study delves into the relationship between institutional quality and energy production within the context of BRI countries. Institutional quality encompasses the effectiveness and efficiency of governance systems, including transparency, accountability, the rule of law, and political stability, which have been widely acknowledged as influential factors in economic and environmental outcomes. Prior research has demonstrated the significant impact of institutional quality on environmental results, with robust institutions facilitating the implementation and enforcement of environmental regulations, thereby reducing environmental degradation and fostering sustainability. Furthermore, institutional quality plays a pivotal role in attracting investments and driving technological advancements, particularly in the renewable energy sector. The study expands on existing literature by specifically examining the link between institutional quality and energy production, with a particular emphasis on electricity generated from various sources such as oil, gas, coal, and renewable energy (excluding hydroelectricity), reflecting the diverse energy mix prevalent in BRI countries. Theoretical frameworks such as Institutional Theory and Environmental Governance provide a theoretical foundation by highlighting the influence of institutional factors, including regulatory frameworks, property rights, and governance structures, on decision-making and behaviors related to energy production. By investigating the association between institutional quality and energy production within BRI countries, the study aims to contribute valuable insights to the field of sustainable development and energy transitions, shedding light on how institutional factors influence energy source choices, the adoption of renewable energy technologies, and the overall environmental sustainability of BRI countries.

## Data and methodology

### Variable description and descriptive statistics

The main purpose of this study is to investigate the impact of different types of electricity production sources such as oil, gas, coal, and renewables and the quality of institutions on the level of CO_2_ emissions for BRI countries that have different income levels. This study divided BRI countries into three income groups: high, middle, and low, following the classification by [54]. The list of countries in each income group is provided in S1 Appendix. The description of the variables used in this study is presented in Table 1.

**Table 1:** Variable description.

Variables	Description	Symbols	Sources
CO_2_ Emissions	Carbon dioxide emission (metric tons per capita)	CO_2_	WDI
Institutional Quality	Government Effectiveness	IQ	WGI
Electricity produced from Oil	% of total production	EPOil	WDI
Electricity produced from Natural Gas	% of total production	EPGas	WDI
Electricity produced from Coal	% of total production	EPCoal	WDI
Electricity produced from Renewables	% of total production	EPRenew	WDI

The data selected for analysis offers several justifications for its use. It provides a comprehensive measurement of institutional quality with government effectiveness, electricity production from oil, gas, and coal measured as a percentage of total production, while renewable electricity production is measured as a percentage of total electricity generated from renewable sources (excluding hydroelectricity), following the methodology established by [55]. The data covers 101 countries participating in the BRI from 2000 to 2020, ensuring a broad representation of the BRI region. The inclusion of a country list in the S1 Appendix enhances transparency and replicability. The data sources, World Governance Indicators (WGI) 2021 and World Development Indicators (WDI) 2021, are reputable and widely recognized. Table 2 shows the results of descriptive statistics and correlation matrix of all variables. The statistical properties of the variables are suitable for estimation. The correlation matrix shows a possible expected relationship between CO_2_ emissions and the energy production from EPGas, EPOil, EPCoal, EPRenew, and IQ. The table reports the minimum values, maximum values, mean, median and values of the first quantile and third quantile. The correlation matrix indicates that the correlation between all the independent variables is roughly between 0.1 and 0.3. If the correlation between the variables is low, multicollinearity will be less of a problem.

**Table 2:** Descriptive statistics.

	CO_2_	IQ	EPOil	EPGas	EPCoal	EPRenew
**Min**	1.385	-2.280	-0.506	0.000	0.000	0.000
**1**^**st**^ **Quantile**	5.270	0.702	0.405	0.000	0.000	0.000
**Mean**	12.136	0.116	17.155	29.050	15.900	2.973
**Median**	7.877	0.212	2.903	15.130	0.000	0.158
**3**^**rd**^ **Quantile**	16.701	0.448	22.206	50.210	22.70	2.302
**Max**	67.312	2.440	100.00	124.90	100.00	48.275
**CO**_**2**_	1					
**IQ**	0.425*	1				
**EPOil**	0.114*	0.110*	1			
**EPGas**	0.447*	0.140*	-0.290*	1		
**EPCoal**	0.022	0.150*	-0.250*	-0.320*	1	
**EPRenew**	-0.083*	0.252*	-0.021	-0.138*	-0.026	1

Note

***, **, and * indicates the significance level at 10%, 5%, and 1% respectively

### Panel quantile regression model

To achieve the objective of this study we used panel quantile fixed effect model. The panel regression analysis was developed by [56]. The innovation in our Panel Quantile Regression (PQR) model lies in its ability to analyze panel data while focusing on quantiles of the conditional distribution. This approach allows for a comprehensive examination of the relationships between variables, capturing potential heterogeneity and providing insights across different quantiles. This innovative aspect of the model makes it particularly suitable for the research paper at hand. The fit of the PQR model for the paper stems from the research objectives and the nature of the data being analyzed. The paper aims to explore the relationship between institutional quality and various energy production sources within the Belt and Road Initiative (BRI) countries. By using panel data, the model can effectively account for individual-specific effects and time-varying factors, providing robust estimates and a more accurate understanding of the relationship between institutional quality and energy production across different quantiles.

Compared to other models, the PQR model offers distinct advantages. Firstly, it allows for non-linear estimation, which is especially valuable when analyzing complex relationships and interactions between variables. This flexibility enables the model to capture more nuanced effects that may not be captured by linear models or models that focus solely on the mean. Secondly, the PQR model takes into account the entire distribution of the response variable, providing insights at different quantiles. Additionally, the PQR model’s ability to handle panel data is a significant advantage. By considering individual-specific effects and accounting for correlation among observations, the model accounts for heterogeneity and time-dependent factors, leading to more accurate and reliable estimates. Overall, the PQR model stands out as the best fit for the research paper due to its innovative approach, suitability for panel data analysis, flexibility in capturing non-linear relationships, and the comprehensive analysis it provides across different quantiles. Its ability to handle panel data and offer insights beyond the mean sets it apart from other models, making it a valuable and diverse tool for investigating the relationship between institutional quality and energy production in the BRI countries.

By using the panel quantile regression technique, the determinants of carbon emissions across the conditional distribution can be estimated, especially for countries where emissions are highest and lowest [57]. The quantile regression is more robust when outliers exist and the random error term does not have a normal distribution [58]. Researchers such as [59–62] have used panel quantile regression to analyze panel data. The following panel quantile regression equation is used in this study:

$$Q_{{yit}^{.}}(\tau ._{k^{.}}|\alpha_{i^{.},}x_{{it}^{.}})=\alpha_{i^{.}}+{x\prime }_{{it}^{.}}{\beta (\tau ._{k^{.}})}_{.},i=1,..,N,t=1,..,T.$$ (1)

In the equation above, *αi* represents an unobservable separate effect, and countries and time have been represented by *i* and *t*. The *x*_*it*_ covariate effect is permitted to relay on the quantiles of time of interest. The drawback of fixed effects is that when many fixed effects are used together, the accidental parameters problem may occur [63, 64]. The estimators will be inconsistent when the population is unbounded, however, the observations for every cross-section stay constant. The basis for these kinds of models is that the probabilities are linear operators [59, 62] gives a method that says that parameters must be evaluated simultaneously with covariate effects for multiple quantiles, by solving the problem of minimization. The difficulty of calculating a large number of parameters is addressed with this approach; the equation for computed parameters is as follows:

$$\underset{\left(\alpha ,\beta \right)}{\min}\overset{K}{\underset{k=1}{\sum }}\overset{T}{\underset{t=1}{\sum }}\overset{N}{\underset{i=1}{\sum }}w_{k}\rho \tau_{k}(y_{it}-\alpha_{i}-x_{it}^{T}\beta (\tau_{k}))+\lambda \overset{N}{\underset{i}{\sum }}|\alpha_{i}|,$$ (2)

Different indexes have been represented in the above equation, for instance, *K*, *T*, and *N* represent the quantile index, the observation index per country, and *i* as nation index (N) respectively. *X*, *ρ*_*τk*_, *and wk* explain the explanatory variables matrix, the linear quantile loss function, and the relative weight of kth quantile, which is the contribution of kth quantile in the estimation of fixed effect, respectively. The parameter *λ* is used to enhance the estimate of the *β* value by bringing individual effects to zero. In this study, we utilize identical weight quantiles (wk = 1/K), following [65]. If the term *λ* is set to zero, the penalty term disappears and the usual fixed effects estimator is retained. We obtain a model estimate without individual effects if the term *λ* extends to infinity. This study used *λ = 1* [66]. The conditional quantile function is defined as follows:

$$Q_{{yit}^{.}}(\tau |\alpha_{i^{.},}\xi_{t^{.},}x_{{it}^{.}})=\alpha_{i^{.}}+\xi_{t^{.}}{+\beta }_{{1\tau }^{.}}{IQ}_{{it}^{.}}{+\beta }_{{2\tau }^{.}}{EPGas}_{{it}^{.}}{+\beta }_{{3\tau }^{.}}{EPOil}_{{it}^{.}}{+\beta }_{{4\tau }^{.}}{EPCoal}_{{it}^{.}}{+\beta }_{{5\tau }^{.}}{EPRenew}_{{it}^{.}}$$ (3)

The emissions indicator is *y*_*it*_. Variable descriptions and descriptive analyses are provided in the next section.

### Results and discussion

A detailed explanation of empirical analysis is given under this heading. To check the association among variables under study, different econometric tests have been used. Furthermore, the empirical results are thoroughly explained and discussed with references. Before using dynamic panel quantile regression, we established the stationarity of each variable. Table 3 provides unit root testing results and it is found that all variables taken in the study are stationary at 1^st^ difference.

**Table 3:** Results of panel unit-root tests.

Variables	Stats	Levels				1^st^ Difference			
		IPS	LLC	Fisher-PP	Fisher-ADF	IPS	LLC	Fisher-PP	Fisher-ADF
**CO**_**2**_	Values	1.484	-3.358	187.160	163.817	-23.222	-23.31	2552.41	901.320
	Prob	0.913	0.004	0.757	0.971	0.000	0.000	0.000	0.000
**IQ**	Values	-3.736	-7.047	299.115	469.971	-21.970	-17.753	2203.23	854.345
	Prob	0.000	0.000	0.000	0.000	0.000	0.000	0.000	0.000
**EPOil**	Values	-12.00	-33.73	731.250	579.306	-24.818	-35.380	3036.00	1093.31
	Prob	0.000	0.000	0.000	0.000	0.000	0.000	0.000	0.000
**EPGas**	Values	-2.234	-5.086	199.134	198.470	-20.303	-19.428	2002.05	691.134
	Prob	0.013	0.000	0.012	0.012	0.000	0.000	0.000	0.000
**EPCoal**	Values	-0.986	-4.122	107.740	117.638	-17.995	-19.303	2030.01	505.651
	Prob	0.162	0.000	0.435	0.206	0.000	0.000	0.000	0.000
**EPRenew**	Values	3.420	-1154	103.860	85.168	-17.330	-16.336	2320.70	576.742
	Prob	0.997	0.124	0.997	1.000	0.000	0.000	0.000	0.000

The findings of the panel cointegration are used to determine a long-run equilibrium relationship between the dependent variable and the independent variables are shown in Table 4. According to the results of co-integration, the null hypothesis has been rejected at a 1% level of significance, as a result there is a co-integration exist among all the explanatory variables and CO_2_ emission in the region.

**Table 4:** Resutls of panel cointegration tests.

Hypothesized No. of CE(s)	Fisher Stat.* (from trace test)	Prob.	Fisher Stat.* (from the max-eigen test)	Prob.
**None**	1840.040	0.000	911.700	0.000
**At most 1**	1147.002	0.000	781.253	0.000
**At most 2**	548.402	0.000	417.833	0.000
**At most 3**	245.301	0.000	200.039	0.000
**At most 4**	120.124	0.004	110.823	0.030

* Probabilities are computed using asymptotic Chi-square distribution.

Table 5 presents the findings of the panel quantile regression analysis, providing insights into the relationship between the independent variables and CO_2_ emissions within the context of the study market. The results indicate that, with the exception of renewable energy production, all independent variables have a positive impact on CO_2_ emissions. Specifically, the effect of IQ on CO_2_ emissions is statistically significant and positive. This finding contrasts with prior research, which has often highlighted a negative relationship between institutional quality and environmental outcomes. However, in the context of the BRI region, the positive relationship suggests that higher institutional quality is associated with higher carbon emissions. Analyzing the coefficient values across different quantiles reveals interesting patterns. Higher institutional quality may result in more CO_2_ emissions for a number of reasons, as shown by the study’s results. These causes may be traced to a number of dynamics and circumstances. Higher institutional quality may sometimes provide a more hospitable climate for certain companies, including those that produce greater carbon emissions. Economic development and growth are often correlated with institutions of higher quality. There is often a rise in the need for energy and resources to sustain industrial activity and satisfy the requirements of a rising population as economies and nations expand. Higher energy use and related carbon emissions may result from this. The positive coefficient of IQ is consistent across all quantiles, ranging from 1.90 at the 10th quantile to 2.01 at the 90th quantile. This indicates that a 1% increase in institutional quality results in a respective increase of 1.9% and 2.1% in CO_2_ emissions. This finding contrasts with previous studies, such as the research conducted by [67, 68], which demonstrated that higher institutional quality promotes environmental sustainability by providing greater freedom and liberty to individuals and businesses.

**Table 5:** Results of panel quantile regression.

Quantile regression	Variables				
	IQ	EPgas	EPoil	EPcoal	EPrenew
10^th^	1.905***	0.014**	0.003***	0.007***	-0.003***
20^th^	1.951***	0.018**	0.003***	0.013***	-0.012***
30^th^	2.201***	0.021**	0.007***	0.034**	-0.029***
40^th^	2.304***	0.024***	0.008***	0.049***	-0.040***
50^th^	2.451***	0.036***	0.009***	0.052***	-0.050***
60^th^	2.481**	0.066***	0.013**	0.055**	-0.059***
70^th^	2.330***	0.122***	0.015**	0.062**	-0.067***
80^th^	2.044***	0.180***	0.022**	0.066***	-0.072**
90^th^	2.017***	0.250***	0.020**	0.073***	-0.066***

Note

***, ** and * are level of significance 1, 5 and 10% respectively

Furthermore, the coefficient values of EPOil, EPGas, and EPCoal demonstrate a positive and statistically significant effect on CO_2_ emissions. This suggests that these specific energy sources contribute to environmental degradation. These findings align with prior research that has consistently highlighted the environmental consequences associated with the use of fossil fuel-based energy sources. The panel quantile regression analysis reveals important insights regarding the relationship between the independent variables and CO_2_ emissions. The positive impact of institutional quality on CO_2_ emissions contrasts with prior findings, emphasizing the unique context of the BRI region. Additionally, the positive and significant effects of EPOil, EPGas, and EPCoal underscore the role of these energy sources in contributing to environmental degradation.

The results of the panel quantile regression analysis demonstrate varying effects of different energy sources on CO_2_ emissions. The coefficient values exhibit an increasing trend as the quantiles increase. For EPGas, the coefficient values in the 10th and 90th quantiles are 0.014 and 0.250, respectively. This suggests that a 1% increase in energy production from natural gas sources leads to a corresponding increase in CO_2_ emissions by 0.014% and 0.250%. These findings indicate that higher energy production from gas is associated with higher carbon dioxide emissions.

Similarly, the coefficient values for EPCoal and EPOil are positive across all quantiles. In the 10th quantile, a 1% increase in oil production leads to a 0.003% rise in CO_2_ emissions, while in the 90th quantile, the increase in CO_2_ emissions amounts to 0.020%. For energy production from coal, the CO_2_ emissions increase by 0.007% in the 10th quantile and 0.073% in the 90th quantile, following a 1% increase in production. These results further emphasize that electricity generated from coal, oil, and gas sources contributes to environmental harm by elevating CO_2_ emissions. Conversely, the findings differ for EPRenew. The analysis reveals a significant negative effect of renewable energy production on CO_2_ emissions. The coefficient value is statistically significant at a 5% level, except for the 10th quantile. A 1% increase in renewable energy production corresponds to a decrease in CO_2_ emissions by 0.003% and 0.066% in the 10th and 90th quantiles, respectively. This signifies that an increased share of energy produced from renewable sources contributes to a reduction in carbon emissions, highlighting the environmentally beneficial nature of renewable energy. The analysis reveals that electricity generated from coal, oil, and gas sources has detrimental effects on the environment, increasing CO_2_ emissions by 0.073%, 0.020%, and 0.250%, respectively. Conversely, the production of one unit of energy from renewable sources leads to a decrease in CO_2_ emissions by 0.003%. These findings underscore the importance of transitioning towards renewable energy sources for mitigating carbon emissions and promoting environmental sustainability within the study market.

**Fig 6:**
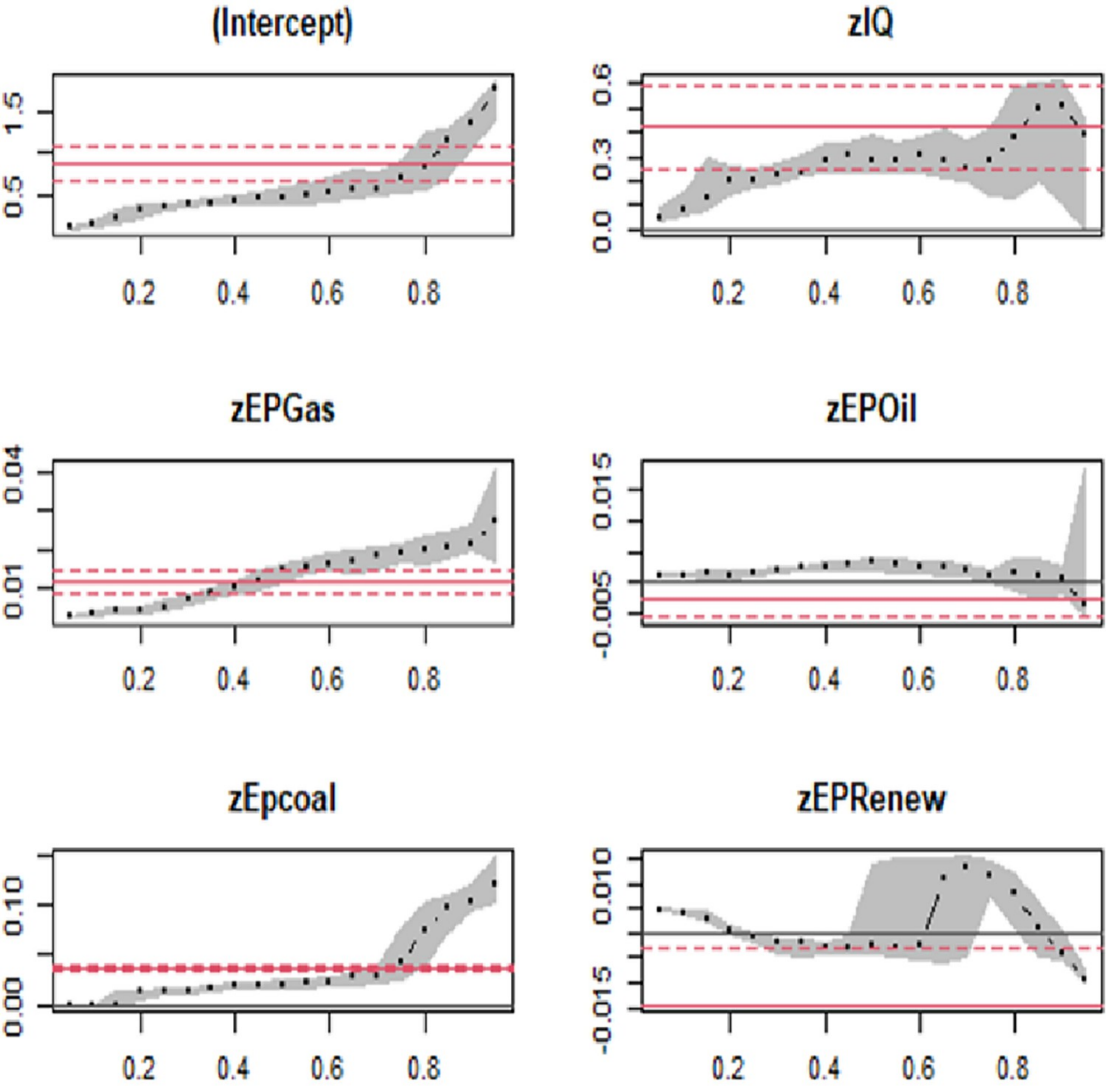
Changes in panel quantile regression coefficients in high-income countries.

Table 6 and Fig 6 show the detailed results for high income countries. The results indicate that higher institutional quality leads to higher carbon emissions. As shown in the table and in the figure, the coefficient of IQ at the 10th quantile is positive and equal to 0.170, while at the 90th quantile, the coefficient value is signific but negative and equal to -3.421. This means that a 1% increase in IQ will increase CO_2_ emission by 0.170%, while at the 90th quantile, a 1% increase in IQ will decrease CO_2_ emission by 3.421%. A possible reason for this negative effect in the future is that IQ can promote green innovation and growth. For example, when institutional quality is high, green innovation has a stronger effect in reducing CO_2_ emissions (Yuan et al., 2021). Moreover, the coefficient values of EPGas, EPOil, and EPCoal show a positive and significant impact on carbon emission. It can be seen in the table that the value of the coefficient increases as the quantiles increase. The coefficient values of EPGas at the 10th and 90th quantiles are 0.091 and 0.245, respectively. This implies that a 1% increase in energy production from gas will increase carbon emissions by 0.091% and 0.245%, respectively. The coefficients for both EPOil and EPCoal are statistically positive in all the quantiles. A 1% increase in EPCoal will result in an increase in emissions by 0.084% at the 10th quantile and by 0.004% at the 90th quantile. For EPOil, a 1% increase in energy production from oil will lead to an increase in emissions by 0.005% at the 10th quantile and by 0.028% at the 90th quantile.

**Table 6:** Results of panel quantile regression for high-income nations.

Quantile Regression	Variables				
	IQ	EPGas	EPOil	EPCoal	EPRenew
10^th^	0.170***	0.091***	0.050***	0.084**	-0.003**
20^th^	0.550***	0.114***	0.043**	0.078***	-0.130***
30^th^	1.670***	0.145**	0.036***	0.073***	-0.143***
40^th^	2.104***	0.161***	0.024**	0.061***	-0.170***
50^th^	1.403*	0.204***	0.020**	0.054***	-0.202***
60^th^	1.240***	0.178***	0.014**	0.046***	-0.211***
70^th^	0.709***	0.207***	0.015**	0.041*	-0.197***
80^th^	-0.836***	0.230***	0.004**	0.008**	-0.235***
90^th^	-3.421**	0.245***	0.028***	0.041***	-0.325**

Note

***, ** and * are level of significance 10, 5 and 1% respectively

On the contrary, contrasting the findings with prior research, the results reveal a distinct pattern for renewable energy sources. The study demonstrates a significant and negative relationship between renewable energy production and CO_2_ emissions. This suggests that as the production of renewable energy increases, carbon emissions decline. Specifically, the results indicate that a 1% increase in renewable energy production leads to a decrease in CO_2_ emissions by 0.003% in the 10th quantile and by 0.325% in the 90th quantile. These findings emphasize the crucial role of transitioning from non-renewable energy sources to renewable sources in environmental preservation, particularly in high-income countries. These results align with previous studies highlighting the environmental benefits associated with greater reliance on renewable energy for electricity generation.

**Fig 7:**
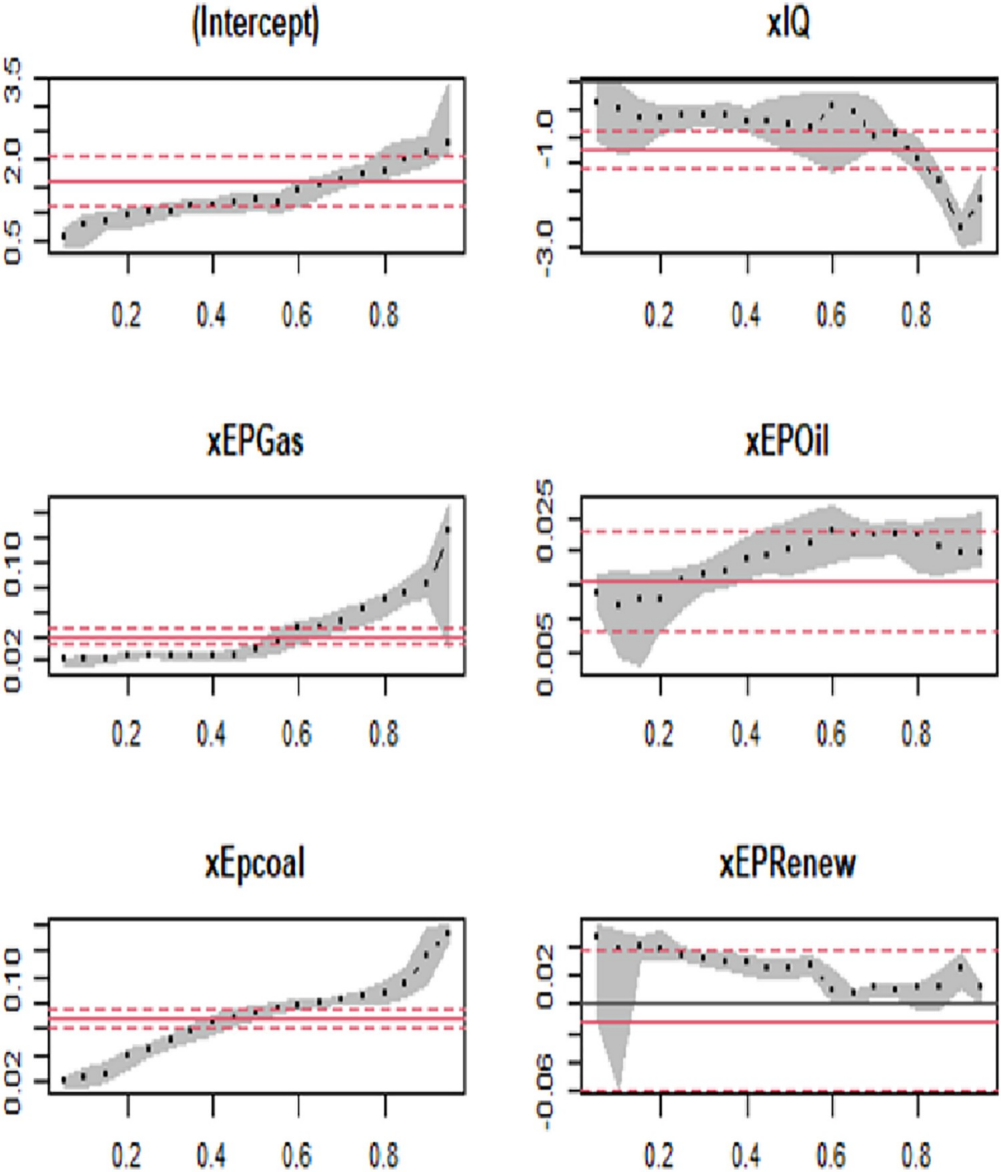
Changes in panel quantile regression coefficients in middle-income countries.

Table 7 and Fig 7 present the results of quantile regression analysis conducted on middle-income countries participating in the BRI. The findings reveal a positive impact of variables EPGas, EPOil, and EPCoal on CO_2_ emissions, indicating that increased energy production from these sources contributes to higher carbon emissions. In contrast, IQ and EPRenew demonstrate a significant and negative influence on carbon emissions and the results are in line with [4, 10, 28, 29]. The negative coefficient of IQ suggests that a 1% increase in institutional quality leads to a 0.502% decrease in CO_2_ emissions in the 10^th^ quantile and a substantial 2.710% decrease in the 90^th^ quantile. This highlights the importance of effective governance and regulatory frameworks in middle-income BRI countries to mitigate CO_2_ emissions. Additionally, the study reveals that increasing the proportion of renewable energy sources has a significant negative impact on CO_2_, supporting the transition towards sustainable energy practices. These findings contribute to a deeper understanding of the dynamics in middle-income BRI countries, emphasizing the crucial role of institutional quality and renewable energy production in reducing carbon emissions.

**Table 7:** Results of panel quantile regression for middle-income nations.

Quantile Regression	Variables				
	IQ	EPGas	EPOil	EPCoal	EPRenew
**10**	-0.502**	0.021**	0.012***	0.022*	-0.060***
**20**	-0.655***	0.022***	0.013***	0.040***	-0.037***
**30**	-0.634***	0.024***	0.017***	0.051***	-0.036**
**40**	-0.706***	0.024**	0.021***	0.066***	-0.030**
**50**	-0.767***	0.028***	0.022***	0.072***	-0.024***
**60**	-0.432***	0.050***	0.023***	0.078***	-0.010***
**70**	-0.988***	0.052***	0.022***	0.103***	-0.011*
**80**	-1.403*	0.071***	0.023***	0.089*	-0.011***
**90**	-2.710***	0.081***	0.021***	0.120***	-0.023***

Note

***, ** and * are level of significance 10, 5 and 1% respectively

Furthermore, building upon prior research, the results indicate a consistent pattern with regards to the impact of non-renewable energy production on CO_2_ emissions in middle-income countries. The coefficients of EPGas, EPOil, and EPCoal reveal a positive relationship, highlighting that an increase in non-renewable energy sources leads to higher carbon emissions. Specifically, a 1% increase in gas-based energy production is associated with a rise in CO_2_ emissions by 0.027% and 0.081%. Similarly, a 1% increase in oil-based energy production leads to an increase in CO_2_ emissions by 0.012% and 0.020% in the 10^th^ and 90^th^ quantiles, while a 1% increase in coal-based energy production results in a rise in CO_2_ emissions by 0.022% and 0.120%.

Contrasting these findings, the study highlights the importance of transitioning to renewable energy sources as a means to mitigate carbon emissions. The results indicate that a 1% increase in renewable energy production is associated with a decrease in CO_2_ emissions by 0.061% and 0.023% in the 10^th^ and 90^th^ quantiles, respectively. These findings align with prior research emphasizing the role of renewable energy in curbing carbon emissions and promoting sustainability. The study’s findings provide further evidence of the detrimental impact of non-renewable energy sources on carbon emissions in middle-income countries. They underscore the urgent need for policy interventions that prioritize a shift towards renewable energy production to combat environmental challenges and foster sustainable development.

**Fig 8:**
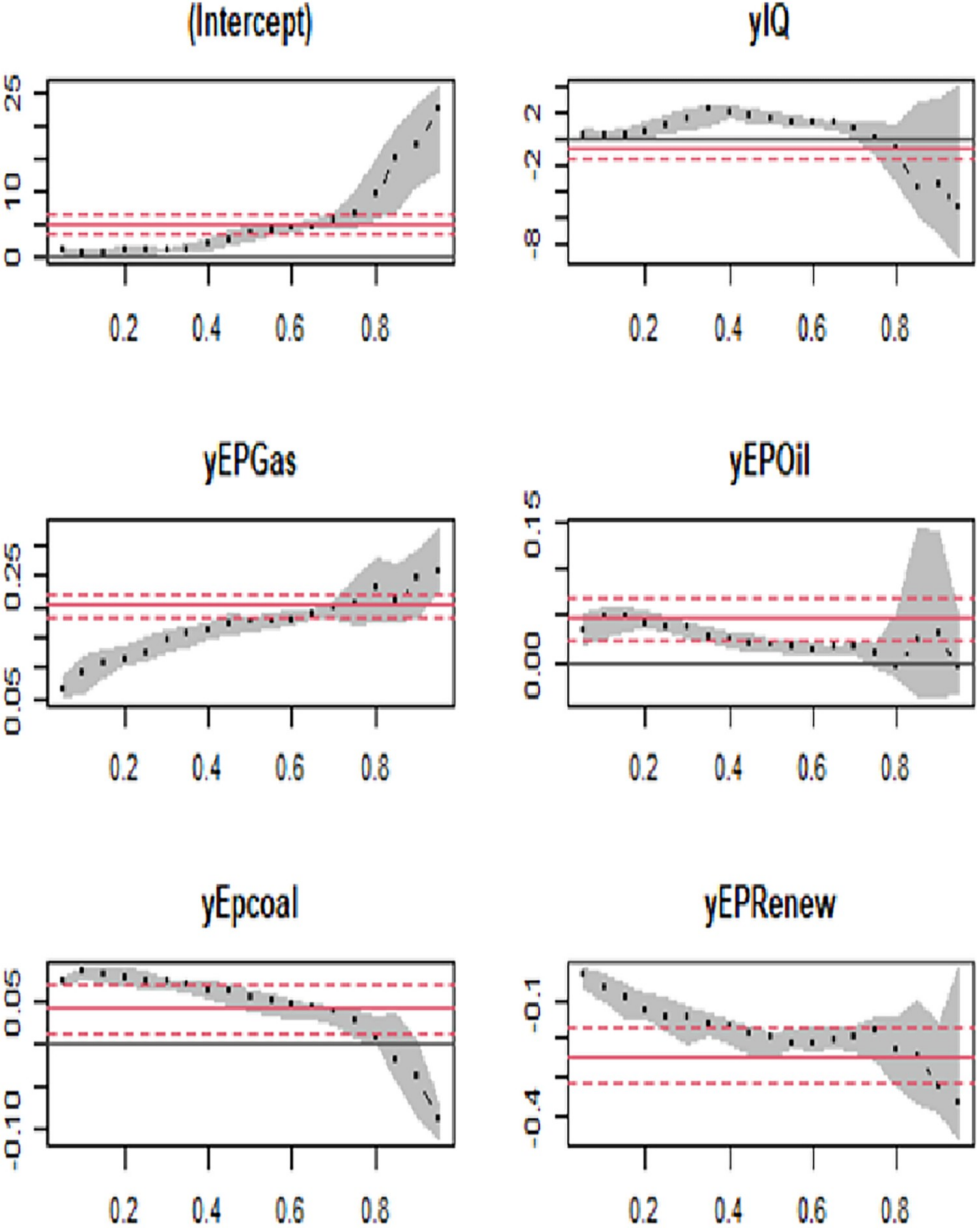
Changes in panel quantile regression coefficients in low-income countries.

The findings of the quantile regression analysis for low-income countries in Table 8 and Fig 8 are align with those of middle-income countries, showcasing some similarities. Consistent with prior research, the results demonstrate a significantly negative effect of IQ on CO_2_ emissions. The coefficient of IQ in the 10^th^ quantile is -0.001, and in the 90^th^ quantile, it is -0.006. This suggests that an improvement in political rights and freedom of information, driven by enhanced institutional quality, can raise public awareness and potentially contribute to lower CO_2_ emissions in low and middle-income economies, as indicated by [69].

**Table 8:** Results of panel quantile regression for low-income nations.

Quantile Regression	Variables				
	IQ	EPGas	EPOil	EPCoal	EPRenew
**10**	-0.001***	0.042***	0.024***	0.012**	-0.001**
**20**	-0.001***	0.033***	0.011***	0.011**	-0.002***
**30**	-0.001***	0.024***	0.010**	0.010***	-0.016***
**40**	-0.002***	0.023***	0.010***	0.009***	-0.020***
**50**	-0.002***	0.020***	0.008**	0.008***	-0.021***
**60**	-0.003***	0.016***	0.007**	0.007***	-0.021***
**70**	-0.003***	0.012***	0.007**	0.006**	-0.035***
**80**	-0.004***	0.007***	0.006**	0.006**	-0.047***
**90**	-0.006***	0.003***	0.006**	0.005**	-0.054***

Note

***, ** and * are level of significance 10, 5 and 1% respectively

In contrast, the coefficients for EPGas exhibit a positive effect on carbon emissions. In the 10^th^ quantile, the coefficient value is 0.042, while in the 90^th^ quantile, it is 0.003. Similarly, all quantiles show positive coefficients for EPCoal and EPOil, indicating that a 1% increase in energy production from oil or coal can lead to corresponding increases in CO_2_ emissions. Specifically, a 1% increase in oil-based energy production results in CO_2_ emission increases of 0.024% and 0.006% in the 10^th^ and 90^th^ quantiles, respectively. Likewise, a 1% increase in coal-based energy production leads to CO_2_ emission increases of 0.011% and 0.005% in the 10^th^ and 90^th^ quantiles, respectively.

Interestingly, the coefficient for EPRenew demonstrates a significant and negative effect on carbon emissions. This indicates that as EPRenew increases, CO_2_ emissions decrease. The results reveal reductions of 0.001% and 0.054% in CO_2_ emissions when EPRenew increases by 1% in the 10^th^ and 90^th^ quantiles, respectively. These findings align with existing literature, highlighting the potential of renewable energy sources in mitigating carbon emissions and promoting environmental sustainability.

The results of the quantile regression analysis for low-income countries reinforce the impact of institutional quality, non-renewable energy production, and renewable energy sources on CO_2_ emissions. These findings contribute to the growing body of evidence emphasizing the importance of effective institutions and the transition to renewable energy as key drivers for achieving sustainable development goals and reducing carbon emissions.

### Pairwise Dumitrescu and Hurlin panel causality test

The above analysis shows that the variables have a long-run relationship, but this does not imply causation. To determine the causal direction for BRI economies, we use a heterogeneous panel causality test that is based on the Granger causality test and was proposed by Dumitrescu and Hurlin. This test can handle both short-run and long-run panel data and account for heterogeneity using W-bar and Z-bar statistics [70].

As shown in Table 9, the p-values of institutional quality and CO_2_ emissions are statistically significant, indicating that they have a causal relationship in both directions. This means that improving institutional quality can reduce CO_2_ emissions and vice versa. Another pair of variables that have a bidirectional causality is EPRenew and CO_2_ emission, as confirmed by their low p-values. This implies that increasing the share of renewable energy can lower CO_2_ emission and reducing CO_2_ emission can encourage the use of renewable energy. The results also demonstrate a one-way causality from EPOil, EPCaol, EPGas to CO_2_, meaning that these fossil fuels contribute to CO_2_ emission but not the other way around.

**Table 9:** Pairwise Dumitrescu and Hurlin panel causality test results.

Null Hypothesis	w-bar stat	z-bar stat	p-value	Decision
IQ ≠ cause CO_2_	-6.823	-3.834	0.002***	IQ ↔ CO_2_
CO_2_ ≠ cause IQ	5.675	2.992	0.003***	
EPOil ≠ cause CO_2_	6.831	3.723	0.000***	EPOil→ CO_2_
CO_2_ ≠ cause EPOil	1.986	0.0255	0.969	
EPGas ≠ cause CO_2_	4.248	4.278	0.039**	EPGas → CO_2_
CO_2_ ≠ cause EPGas	1.394	0.453	0.980	
EPCoal ≠ cause CO_2_	6.898	8.346	0.000***	EPCoal → CO_2_
CO_2_ ≠ cause EPCoal	2.114	2.928	0.400	
EPRenew ≠ cause CO_2_	-7. L33	-4.765	0.086*	EPRenew ↔ CO_2_
CO_2_ ≠ cause EPRenew	3.831	1.937	0.029**	

Note

***, ** and * are level of significance 1, 5 and 10% respectively. ↔ indicate bidirectional causality, → represent unidirectional causality.

## Conclusion and policy suggestions

This study contributes to the existing literature by examining the relationship between CO_2_ emissions, institutional quality, and disaggregated energy production sources in BRI countries from 2000 to 2020. The BRI countries were categorized into three groups based on income level: high, middle, and low-income. Analytical techniques such as IPS, LLC unit-root test, Johansen & Fisher co-integration, and panel quantile regression were employed to provide empirical evidence and insights into the research variables. The panel quantile regression analysis captures heterogeneities in distributions and unobserved individual effects, offering a comprehensive understanding of the relationships.

The empirical analysis establishes a long-term relationship between institutional quality, energy production from coal, gas, oil, renewables, and carbon emissions in both the overall BRI countries and the income-wise disaggregated groups. The variables were found to be stationary at the first difference, and panel co-integration results confirm a long-term relation between these variables. Specifically, the study reveals a positive association between CO_2_ emissions and institutional quality. Higher institutional quality in the BRI region may provide more freedom and liberty, benefiting the public and businesses. However, the relationship differs slightly for middle- and low-income countries, potentially influenced by the role of political rights and freedom of information in raising public awareness, as suggested by [69].

Moreover, the analysis demonstrates a positive relationship between carbon emissions and energy production from fossil fuels, including gas, oil, and coal. The combustion of these fuels contributes to smog and air pollution, thereby increasing environmental risks. In contrast, a negative relationship is observed between CO_2_ emissions and energy production from renewable sources. The effect of renewable energy production in income-wise disaggregated BRI countries aligns with the overall BRI region, with a slightly higher impact on CO_2_ emissions in high-income countries, as they already have a significant share of their energy derived from renewable sources. Considering the environmental benefits, global growth, and the ongoing development of smart grids, it is highly likely that renewable energy will become the dominant source of energy in the future.

This study emphasizes the significance of improving institutional quality in Belt and Road Initiative countries to effectively address environmental degradation. By implementing appropriate laws and regulations, governments can utilize institutional quality to lower CO_2_ emissions and improve environmental conditions. Policymakers should prioritize the revision of existing policies to incorporate measures targeting air pollution, safe drinking water, and clean water. High-income countries should reevaluate their policies related to law and order, political rights, and freedom of information to raise environmental awareness. Meanwhile, middle- and low-income countries should focus on enhancing institutional quality to improve environmental quality and reduce CO_2_ emissions. Despite the challenges faced by poorer nations, the establishment of fair and robust institutions can facilitate the adoption of effective environmental regulations, promoting sustainable practices and achieving long-term environmental sustainability goals.

The effect on CO_2_ emissions varies slightly among countries. High-income countries, which already utilize renewable sources for a portion of their energy needs, exhibit a greater impact on CO_2_ emissions. Considering the environmental benefits, potential for global cooperation, and advancements in smart grid technology, it is anticipated that renewable energy will dominate the energy sector in the future. Policymakers should provide clear and specific incentives, such as tax reductions and subsidies, for renewable energy investors. It is crucial to establish transparent criteria and standards for assessing the environmental impact and economic feasibility of renewable energy projects. Moreover, fostering collaboration and knowledge sharing among BRI countries will facilitate the transfer and diffusion of renewable energy technology. Supporting the development of local capacity and skills in renewable energy production and usage is also essential to ensure widespread adoption of clean energy sources.

To mitigate the impact of fossil fuel energy production, BRI countries should prioritize energy policies aimed at reducing reliance on fossil fuels and diversifying their energy mix. This necessitates high-income countries investing in middle and low-income countries to bolster their renewable energy production. Such collaborative efforts will significantly contribute to the reduction of CO_2_ emissions in BRI countries. Additionally, national banks should offer favorable loan terms and conditions to commercial banks specifically for investments in green technologies and carbon-free projects. This financial support will incentivize greater investment in environmentally friendly solutions.

Governments should establish clear and stringent environmental standards and regulations to mitigate the negative impacts of energy production and usage. This includes setting emission limits, implementing pollution control measures, and establishing guidelines for environmental impact assessments. Policymakers should ensure effective enforcement of these standards and impose penalties for non-compliance. Furthermore, governments should promote the use of clean technologies and support research and innovation in environmental technologies.

Governments and national banks should provide financial support for renewable energy investments. This can be done through favorable loan terms and conditions, grants, and subsidies specifically targeting renewable energy projects. Financial institutions should also consider incorporating green finance principles and offering financial products that support renewable energy initiatives. Governments should work with banks and financial institutions to develop mechanisms that facilitate access to capital for renewable energy developers and investors. Collaboration and cooperation between BRI countries and international organizations are crucial for achieving a sustainable energy future.

The study has several limitations that should be acknowledged. Firstly, the availability and reliability of data on institutional quality, renewable energy production, and environmental indicators in BRI countries play a crucial role in determining the study’s findings and policy implications. Insufficient data or inconsistencies in data quality across countries can impact the accuracy and generalizability of the study’s results. Secondly, while the study focuses on examining the relationship between institutional quality, renewable energy production, and environmental outcomes in BRI countries, it may not fully capture the complexity and interaction of other factors that could influence these relationships. Socioeconomic factors, cultural contexts, and technological advancements are just a few examples of additional variables that could provide a more nuanced understanding of the dynamics at play. A more comprehensive analysis incorporating these factors would enhance the study’s insights. Lastly, caution should be exercised when extrapolating the findings of this study to other regions. The unique characteristics and contexts of the BRI countries may limit the applicability of the study’s conclusions to regions with different socioeconomic, political, and environmental conditions. Each region may have its own specific challenges and opportunities that warrant separate investigations and tailored policy recommendations.

Future recommendations for research and policy include conducting longitudinal studies to examine the long-term effectiveness of interventions targeting institutional quality, renewable energy generation, and environmental outcomes. Comparative analysis should be employed to learn from best practices and experiences in other regions or countries with similar development paths, adapting them to the specific context of the BRI countries. In-depth case studies within BRI countries can provide valuable insights into country-specific challenges and opportunities related to institutional quality, renewable energy generation, and environmental management. Additionally, research should focus on emerging technologies for energy generation, storage, and efficiency to assess their viability, scalability, and cost-effectiveness, thereby shaping future policies and investments in renewable energy. Implementing these comprehensive future recommendations will contribute to sustainable development and informed decision-making in the BRI countries.

## Supporting information

S1 Appendix Selected BRI countries. (DOCX)
