# Supplementary material for: How institutional quality, and energy production sources, affect the environmental sustainability of bri countries: A comparison of different income groups
Source: PLoS One. 2023 Sep 12;18(9):e0291144. doi: 10.1371/journal.pone.0291144 (PMC10497143; doi:10.1371/journal.pone.0291144)
Supplement: S1 Appendix — (DOCX) [file pone.0291144.s001.docx]

**Appendix A**

**Selected BRI Countries**

| **Country** | **Category** | **Country** | **Category** | **Country** | **Category** |
| --- | --- | --- | --- | --- | --- |
| Austria | High Income | Albania | Middle Income | Benin | Low Income |
| Bahrain | High Income | Algeria | Middle Income | Congo, Dem. Rep. | Low Income |
| Brunei Darussalam | High Income | Angola | Middle Income | Ethiopia | Low Income |
| Chile | High Income | Armenia | Middle Income | Mozambique | Low Income |
| Croatia | High Income | Azerbaijan | Middle Income | Nepal | Low Income |
| Cyprus | High Income | Bangladesh | Middle Income | Niger | Low Income |
| Czech Republic | High Income | Belarus | Middle Income | Senegal | Low Income |
| Estonia | High Income | Bolivia | Middle Income | Tajikistan | Low Income |
| Greece | High Income | Bosnia and Herzegovina | Middle Income | Tanzania | Low Income |
| Hungary | High Income | Botswana | Middle Income | Yemen, Rep. | Low Income |
| Italy | High Income | Bulgaria | Middle Income | Zimbabwe | Low Income |
| Korea, Rep. | High Income | Cambodia | Middle Income |  |  |
| Kuwait | High Income | Cameroon | Middle Income |  |  |
| Latvia | High Income | China | Middle Income |  |  |
| Lithuania | High Income | Congo, Rep. | Middle Income |  |  |
| Luxembourg | High Income | Costa Rica | Middle Income |  |  |
| Malta | High Income | Cote d'Ivoire | Middle Income |  |  |
| New Zealand | High Income | Cuba | Middle Income |  |  |
| Oman | High Income | Dominican Republic | Middle Income |  |  |
| Panama | High Income | Ecuador | Middle Income |  |  |
| Poland | High Income | Egypt, Arab Rep. | Middle Income |  |  |
| Portugal | High Income | El Salvador | Middle Income |  |  |
| Qatar | High Income | Gabon | Middle Income |  |  |
| Trinidad and Tobago | High Income | Georgia | Middle Income |  |  |
| Saudi Arabia | High Income | Ghana | Middle Income |  |  |
| Singapore | High Income | Indonesia | Middle Income |  |  |
| Slovak Republic | High Income | Iran, Islamic Rep. | Middle Income |  |  |
| Slovenia | High Income | Iraq | Middle Income |  |  |
| United Arab Emirates | High Income | Jamaica | Middle Income |  |  |
| Uruguay | High Income | Kazakhstan | Middle Income |  |  |
|  |  | Kenya | Middle Income |  |  |
|  |  | Kyrgyz Republic | Middle Income |  |  |
|  |  | Lebanon | Middle Income |  |  |
|  |  | Libya | Middle Income |  |  |
|  |  | Malaysia | Middle Income |  |  |
|  |  | Moldova | Middle Income |  |  |
|  |  | Mongolia | Middle Income |  |  |
|  |  | Morocco | Middle Income |  |  |
|  |  | Myanmar | Middle Income |  |  |
|  |  | Namibia | Middle Income |  |  |
|  |  | Nigeria | Middle Income |  |  |
|  |  | North Macedonia | Middle Income |  |  |
|  |  | Pakistan | Middle Income |  |  |
|  |  | Peru | Middle Income |  |  |
|  |  | Philippines | Middle Income |  |  |
|  |  | Romania | Middle Income |  |  |
|  |  | Russian Federation | Middle Income |  |  |
|  |  | Serbia | Middle Income |  |  |
|  |  | South Africa | Middle Income |  |  |
|  |  | Sri Lanka | Middle Income |  |  |
|  |  | Sudan | Middle Income |  |  |
|  |  | Thailand | Middle Income |  |  |
|  |  | Tonga | Middle Income |  |  |
|  |  | Tunisia | Middle Income |  |  |
|  |  | Turkey | Middle Income |  |  |
|  |  | Ukraine | Middle Income |  |  |
|  |  | Uzbekistan | Middle Income |  |  |
|  |  | Venezuela, RB | Middle Income |  |  |
|  |  | Vietnam | Middle Income |  |  |
|  |  | Zambia | Middle Income |  |  |
